# A National Assessment of Access to Technology Among Nursing Home Residents: A Secondary Analysis

**DOI:** 10.2196/11449

**Published:** 2019-03-05

**Authors:** Kimberly Ryan Powell, Gregory Lynn Alexander, Richard Madsen, Chelsea Deroche

**Affiliations:** 1 College of Nursing University of Tennessee, Knoxville Knoxville, TN United States; 2 Sinclair School of Nursing University of Missouri, Columbia Columbia, MO United States

**Keywords:** nursing homes, health information technology, patient access, patient portals, personal health records, patient engagement, person-centered care

## Abstract

**Background:**

According to the National Center for Health Statistics, there are over 1.7 million nursing home residents in the United States. Nursing home residents and their family members have unique needs and stand to benefit from using technology empowering them to be more informed and engaged health care consumers. Although there is growing evidence for benefits of patient-facing technologies like electronic patient portals on patient engagement in acute and outpatient settings, little is known about use of this technology in nursing homes.

**Objective:**

The purpose of this study was to report findings from a secondary analysis of data from a national nursing home study of information technology (IT) adoption, called IT sophistication. We describe the extent to which nursing homes (n=815) allow residents or their representatives to access technology including electronic health records, patient portals, and health information-exchange systems as well as the ability of the residents or representatives to self-report data directly into the electronic health record.

**Methods:**

We used descriptive statistics and regression techniques to explore relationships between information technology adoption (IT sophistication) and residents’ or their representatives’ access to technology. Covariates of location, bed size, and ownership were added to the model to understand their potential influence on the relationship between IT sophistication and resident access to technology.

**Results:**

Findings revealed that resident access to technology was a significant predictor of the nursing home IT sophistication (*P*<.001). The inclusion of covariates—nursing home location, bed size, and ownership—with their interactions produced a nonsignificant effect in the model. Residents’ or their representatives’ use of electronic health records and personal health records were both significant predictors of overall IT sophistication (*P*<.001).

**Conclusions:**

As nursing homes continue to progress in technological capabilities, it is important to understand how increasing IT sophistication can be leveraged to create opportunities to engage residents in their care. Understanding the impact of health information technology on outcomes and which technologies make a difference will help nursing home administrators make more informed decisions about adoption and implementation.

## Introduction

Using health information technology (IT) to engage residents and promote person-centered care in nursing homes is a novel idea. Although there is growing evidence of the benefits of health IT on patient engagement in acute and outpatient settings, little is known about the use of this technology in postacute settings like nursing homes. Over 1.7 million nursing home residents in the United States [1] and their family members have unique needs and stand to benefit from using technology that will empower them to be more informed and engaged in their health care.

Nursing homes face unique challenges related to the adoption of IT. Over the last 9 years, the Centers for Medicare and Medicaid Services have spent over US $38 billion on incentivizing the adoption of IT in the United States through the “meaningful use” program [2]. Despite this substantial investment, not all providers were eligible to participate in the incentive program. Long-term acute care hospitals, inpatient psychiatric hospitals, home health agencies, and nursing homes (which provide rehabilitation and skilled nursing services) were excluded from the incentive program and now face substantial challenges in IT adoption. For example, prior to 2008, only 6% of long-term care hospitals had a basic electronic health record (EHR) in place, which is an adoption rate less than half of that among acute care hospitals [3]. In fact, between the years 2008 and 2015, acute care hospitals experienced a more than eight-fold increase in EHR adoption [4]. In 2017, more than 95% of all federal acute care hospitals in the United States had a certified EHR in place [5]. This gap in EHR adoption between acute and postacute providers continues to grow as more sophisticated EHRs are adopted, many of which include opportunities to improve patient engagement by giving patients digital access to their medical records.

Improving patient and family engagement has been identified as a priority for improving health care in the United States by organizations such as the National Academy of Medicine and National Quality Forum [6,7]. The role of patients and their caregivers in health care is changing, as more emphasis is being placed on person-centered care and shared decision making. Person-centered care is emerging as a targeted approach for improvement across diverse settings in health care including nursing homes. Many definitions of person-centered care have been developed; however, at the center of this concept is the importance of incorporating patient needs and perspectives into care delivery. In nursing homes and other postacute settings, person-centered care has been identified as a way to overcome institutionalization and dependency through enhanced autonomy and empowerment of residents and their family members [8]. Improvements in outcomes related to patient engagement such as patient activation and perceived quality of life are beginning to emerge as more emphasis is placed on patient access to their health data via technologies such as health information exchange (HIE) networks, patient portals, and personal health records.

Bidirectional HIE technology can be used to improve resident and family engagement in nursing homes. Electronic HIE networks allow providers, nurses, pharmacists, and patients to access and securely share medical information electronically, contributing to timely, safe, and cost-effective care. Despite the growing availability of secure electronic data exchange, most patients are still relying on paper-based records that they carry from one appointment to the next [9]. Improving the quality of care for nursing home residents requires HIE between a variety of stakeholders. In nursing homes, HIE is used frequently to monitor resident care tasks, coordinate and authorize care plans, communicate about resident care, and manage administrative and financial activities [10]. Without these exchange capabilities, nursing home providers face greater risk of breaks in vital communication about resident care, using incomplete clinical data, and experiencing limited capacity to make informed care decisions [10]. The potential benefit of HIE to nursing home residents includes improved communication among multiple providers, which may result in improved outcomes such as fewer medical errors, improved transitions in care, and reduced avoidable hospitalizations [11]. Although most HIE systems are used exclusively by providers, provision of access to these data to residents or residents’ representatives should be explored as an opportunity to promote engagement and person-centered care.

Promoting patient and family engagement using technological interfaces such as personal health records or patient portals has become a hot topic in health care. Patient portals are Web-based accounts that connect patients to their EHR. These “tethered” (ie, connected to the EHR) portals provide patients and family members with convenient and reliable access to information and offer resources to promote health by facilitating collaborative relationships between patients and providers, granting people access to and allowing them control over their personal health data, and promoting improved engagement in their health care [12]. Typical features of the patient portal include secure access to visit summaries, medication lists, test results, and appointment requests. More advanced functions such as secure messaging, access to educational resources, and the ability for the patient to enter data directly into the EHR are becoming more widely available. Recent empirical studies on patient portals have focused mainly on specific aspects of use (eg, use of specific functions such as secure messaging) and user characteristics, and almost all of these studies have been conducted in primary care and specialty clinics [13]. Although these studies hold promise for engaging patients in acute and outpatient settings, little is known about the use of patient-facing technologies, such as patient portals, by nursing home residents and their family members.

The purpose of this study was to assess resident access to technology in a nationally representative sample of US nursing homes and to explore the relationship between resident access to technology and overall IT adoption, called IT sophistication. The following research questions were used to guide the study:

1. What is the relationship between IT sophistication and nursing homes that have technology available to residents/residents’ representatives?
2. What is the relationship between IT sophistication and nursing homes that have technology available to residents/residents’ representatives after adjusting for type of ownership, bed size, and profit status?
3. How do specific resident-access components (ie, access to the EHR, personal health record, health information exchange, and self-reported data) impact total IT sophistication?

## Methods

### Design

We conducted a secondary analysis of data on the use of technology by residents and residents’ representatives from a national survey of nursing home administrative leaders [14]. Nursing home administrators were chosen to complete the survey themselves or to identify a designee with oversight of IT systems. These administrators or designees were chosen because they had core knowledge of nursing home care processes and acted as managing officers in planning, organizing, directing, and controlling day-to-day operations in their facilities [15]. Nursing homes were randomly selected from each state using the Nursing Home Compare dataset. This publicly available dataset is maintained by the Centers for Medicare and Medicare Services [16]. The recruitment period used in this study was January 1, 2014, through July 31, 2015. This study was approved by the Institutional Review Board at the University of Missouri, Columbia (project number 1209004; exempt application number 116979).

### Measures

We used a survey developed to measure nursing home IT adoption, called IT sophistication. A detailed description of the survey has been previously published elsewhere [14,17,18]. The survey contains a total of 50 questions related to three IT sophistication dimensions (IT capabilities, extent of IT use, and degree of internal and external IT integration) and three domains of care (resident care, clinical support, and administrative activities). The total IT sophistication score is calculated as the sum of responses in each of the three dimensions and three domains. The survey has been tested previously and determined to have good reliability and validity measures [17,19]. The Cronbach alpha values for the IT dimensions among the three clinical domains of resident care, clinical support, and administrative activities are 0.87-0.88, 0.86-0.91, and 0.69-0.80, respectively [14].

If a respondent indicated that they had the capability to offer residents or their representatives access to technology, they were asked to rate the extent to which residents or their representatives use that technology. If the respondent indicated no capability, they were not asked about the extent of IT use. The third dimension of IT sophistication (degree of IT integration) was not relevant to this secondary analysis.

To answer our research questions, we focused on four questions in the survey related to the dimension, extent of use, domain, and resident care. These questions specifically inquire about residents’ or resident representatives’ use of technology (Textbox 1).

Participants were asked to rate the extent of use of technology by residents or residents’ representatives on a scale of 0-6 points, with 0 indicating “not at all” and 6 indicating “very much.” We calculated a cumulative score using data from the questions in Textbox 1 for each home with a minimum score of 0 and maximum score of 24. For example, a nursing home with a total of 18 points could have reported a score of 6 for question 1 (use of EHR), 6 for question 2 (use of personal health record), 6 for question 3 (use of HIE), but 0 for question 4 (self-reported data).

### Sample

The sampling strategy used in the primary study has been published elsewhere [14,18]. The final sample consisted of 815 nursing home leaders from every US state (except for Guam, Puerto Rico, and US Virgin Islands). Nursing homes were not stratified by the characteristics of location (eg, rural/urban), bed size (eg, <60 beds, 60-120 beds, >120 beds), and ownership (eg, for profit/not for profit) prior to random selection in case there was inadequate representation of these characteristics in some states. For example, Wyoming has a total of 38 nursing homes in the state, so there may not be any large homes in rural areas. This approach was used to ensure each facility had an equal opportunity to participate regardless of the characteristics of location, bed size, and ownership.

### Data Analysis

Descriptive statistics were used to characterize the sample by using frequencies, means, and SDs. Simple linear regressions were conducted to examine the univariate relationships between IT sophistication and resident access to technology including access to EHR, patient portal, and HIE network and ability to self-enter data into the EHR. The dependent variable in the analysis was total IT sophistication (total_IT*), and the independent variable was resident access to technology (Res_Tech). Note that the Res_Tech variable makes a direct contribution to the total_IT*. Consequently, to assess the effect of Res_Tech on total_IT*, we decided to construct a new variable, which is total_IT* minus the contribution of Res_Tech. We performed the analysis first using the unadjusted total IT sophistication score and again using the new variable (total_IT*) to see if this changed the results. Ultimately, there was little difference when using the new variable in the analysis, so we decided to proceed with total_IT* as the dependent variable because we considered this to be a more statistically sound approach. We also calculated values of variance inflation factors to check for multicollinearity. In this case, the values were between 1 and 1.5, indicating multicollinearity, and the variance inflation factors were of little concern in the models presented.

**Survey questions related to the extent of use of technology by the residents or residents’ representatives.**

1. Resident or residents’ representative use of electronic health records
2. Resident or residents’ representative use of personal health records
3. Resident or residents’ representative use of health information exchange
4. Resident or residents’ representative use of self-reported data into an electronic health record

Next, covariates of location, bed size, and ownership were added to the model to understand their potential influence on the relationship between IT sophistication and resident access to technology. Lastly, we examined four components of Res_Tech—residents’ or their representatives’ use of EHRs, use of personal health records or the patient portal, use of health information exchange, and ability to enter self-reported data into the EHR—to understand their unique contribution to the overall IT sophistication score. SAS software, version 9 (SAS Institute Inc, Cary, NC) was used for all analyses.

## Results

Of the 815 nursing homes included in this sample, 702 had a total Res_Tech score of 0, indicating no resident access to technology. Despite having a total Res_Tech score of 0, several of these homes had very high total_IT* sophistication (range 24.8-716.3; mean 282.6; SD 127.7). Sixteen homes in our sample had total Res_Tech scores of ≥12, indicating that these homes have a high extent of use of technology by residents or residents’ representatives. Demographic characteristics of the sample as well as those with a Res_Tech score of ≥12 are described in Table 1.

To address the first research question, we examined the contribution of Res_Tech to total_IT* sophistication (scored on a scale of 0 to 24 points). Table 2 shows the weighted means of total_IT* for each level of Res_Tech. The mean for each level was found using the Survey Means procedure with a Domain statement. Table 3 shows mean the Res_Tech scores according to nursing homes in the lower 20%, middle 20%, and upper 20% of total_IT* sophistication.

**Figure 1:**
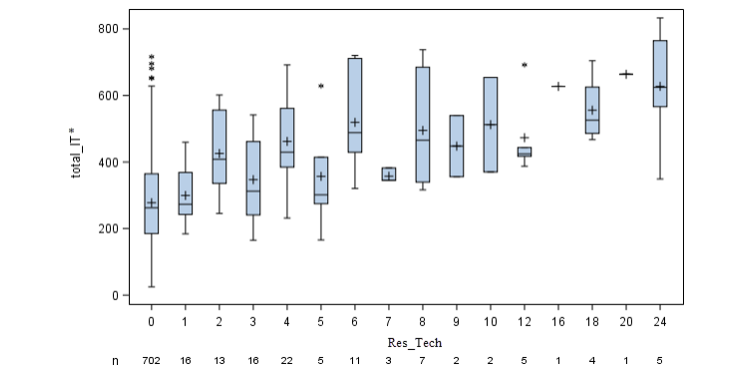
Weighted means of total IT sophistication scores (total_IT*=total_IT–Res_Tech) for each level of resident or resident representative’s access to technology (Res_Tech). Total_IT*: total information technology sophistication; Res_Tech: resident access to technology.

Figure 1 shows skeletal boxplots for the weighted means of total_IT* sophistication scores for each level of the scale. Overall, there appears to be an upward trend in the plot, indicating a positive correlation between total_IT* sophistication and the total Res_Tech score.

We used regression techniques to model the relationship between total_IT* sophistication and Res_Tech scores. We used the SurveyReg procedure to determine if the slope of the fitted regression line was significantly different from 0. It was different from 0, with an estimated slope of 17.7 (*R*^2^=0.15; *F*=94.39; *P*<.001).

In order to address the second research question, we examined the model when the three covariates (nursing home characteristics) of location, bed size, and ownership with their interactions were included in the model. In this case, the estimated slope changed slightly to 17.3 (*R*^2^=0.22; *F*=71.35; *P*<.001) indicating a small effect on including these characteristics in the model (Table 4).

Finally, to address the third research question, we assessed the contribution of each component (questions 1-4) of the Res_Tech score. Using the Total_IT* as the response variable, we fit a regression model using the four questions as predictors. Questions 3 and 4 were not statistically significant. Fitting a model with questions 1 and 2 showed that both were significant predictors (*P*<.001). The estimated coefficients were 29.7 and 29.0, respectively.

**Table 1:** Characteristics of nursing homes completing the survey and those with Res_Tech scores ≥12.

Characteristics		Sample (N=815)	Nursing homes with Res_Tech^a^ scores ≥12
**Ownership, n (%)**			
	For-profit corporation	448 (54.9)	11 (68.7)
	Individual	25 (3.1)	0
	Limited liability	6 (0.7)	0
	Partnership	64 (7.9)	0
	Government	6 (0.7)	0
	Nonprofit	266 (32.7)	5 (45.4)
**Location, n (%)**			
	Metro (population >50,000)	478 (58.7)	11 (68.7)
	Micro (10,000-49,999)	126 (15.5)	3 (18.7)
	Small town (2500-9999)	114 (14)	1 (6.2)
	Rural (<2500)	97 (12)	1 (6.2)
**Number of beds, n (%)**			
	>120 beds	191 (23.4)	5 (45.4)
	60-120 beds	472 (57.9)	8 (56.2)
	<60 beds	152 (18.6)	3 (18.7)
Total IT sophistication score (mean)		299.3	555.8

^a^Res_Tech: resident access to technology scores.

**Table 2:** Means of total IT sophistication for each level of resident access to technology.

Res_Tech^a^ score	n	Mean	Standard error
0	702	282.6	5.7
1	16	300.0	23.3
2	13	417.6	39.1
3	16	312.5	28.1
4	22	460.6	27.5
5	5	283.3	42.0
6	11	491.4	39.0
7	3	352.3	10.6
8	7	479.2	62.1
9	2	439.1	66.7
10	2	526.6	97.8
12	5	467.2	49.3
16	1	613.9	0.0
18	4	493.6	24.4
20	1	646.9	0.0
24	5	665.7	74.4

^a^Res_Tech: Resident access to technology.

**Table 3:** Resident access to technology scores classified by lower 20% (total_IT*≤175.2), middle 20% (40th-60th percentile; total_IT* between 244.6 and 323.8), and upper 20% (total_IT*≥414.7) of total IT sophistication.

Total_IT*^a^ group	Mean	Minimum	25th percentile	Median	75th percentile	Maximum
Lower 20%	0.05	0	0	0	0	5
Middle 20%	0.32	0	0	0	0	8
Upper 20%	2.79	0	0	0	4	24

^a^Total_IT*: total information technology sophistication.

**Table 4:** Model total IT sophistication predicted by resident access to technology (covariates included).

Parameter	Estimate	Standard error	*P* value
Intercept	544.88	0.00	<.001
Res_Tech^a^	17.30	1.95	<.001
Location - metro	–215.89	20.23	<.001
Location - micro	–183.35	33.58	<.001
Location - rural	–166.98	78.86	.04
Location - small town	Reference	Reference	—^b^
Bed size 60-120	–304.47	35.03	<.001
Bed size <60	–275.61	52.60	<.001
Bed size >120	Reference	Reference	—

^a^Res_Tech: Resident access to technology.

^b^Not available.

## Discussion

### Principal Findings and Overview

Through this secondary analysis of national survey data, we examined the extent to which nursing homes allow residents and their representatives to access technology and compared these capabilities to overall IT sophistication. We found nursing homes with higher capabilities for resident access to technology have higher overall IT sophistication. As nursing homes continue to progress in technological capabilities, it is important to understand the impact of IT on outcomes and which technologies make a difference. This understanding will help nursing home administrators make informed decisions about adoption of technology and how it might be used to facilitate resident engagement and promote person-centered care.

The relationship we found between resident access to technology and overall IT sophistication in nursing homes is important for several reasons. First, it is clear that existing technological capabilities for nursing homes span a wide range. On one end, there are homes with highly sophisticated and integrated IT systems; however, they do not extend the use of these systems to residents and their family members. Of the 815 nursing homes included in this analysis, 702 (about 86%) homes had a total Res_Tech score of 0, indicating no resident access to technology. This implies that nursing homes are choosing to make IT investments in other areas rather than in patient-facing technologies. It is not known if this is due to limits in technical capabilities (ie, they do not have proper systems, infrastructure, or knowledgeable workforce to support resident access) or other reasons. In recent studies, providers have expressed concerns about patient-level access to health data, citing security, workflow, and regulatory concerns [20,21]. It is important to note, however, that none of these studies focused specifically on resident access to technology in nursing homes. Further research is needed to understand barriers to resident access that are unique to the nursing home setting and how they might be mitigated.

At the other end of the spectrum are nursing homes with high IT sophistication including some degree of resident access. There were 16 nursing homes in our study that had a Res_Tech score of ≥12. These nursing homes are early adopters of patient-facing technology and should be studied to understand how they are using this technology in the postacute setting. This was the first study of resident access to technology in nursing homes; thus, it establishes an important baseline upon which future work can be built. Future studies should explore the benefits and barriers as well as perceptions of patient-facing technologies in nursing homes and work to leverage these capabilities in a way that is most impactful for resident and family engagement.

In our study, resident access to the EHR and personal health record, or patient portal, was a significant contributor to predicting overall IT sophistication scores. Although nursing homes are ineligible for the Centers for Medicare and Medicaid Services incentive program, EHR adoption in nursing homes is growing. In 2016, the majority (64%) of US nursing homes had a fully implemented and operational EHR [22]. Having an EHR in place creates new opportunities for patient and family engagement, especially through the use of personal health records or patient portals. Although resident access to the EHR via a patient portal may be a new concept for nursing homes, patient-facing access is growing quickly in other sectors. As of 2015, 95% of hospitals in the United States provided patients with the ability to view their health information electronically and 69% allowed patients to view, download, and transmit their health information [23]. Patient portals are in their infancy, and evidence on their use remains largely limited to acute and ambulatory settings. Future research should explore the opportunities for enhanced portal use through training and development of features that residents and their families value and have the potential to improve care.

As EHRs become more mainstream in nursing homes, we can begin to explore their potential benefits such as connecting providers, patients, and other members of the interdisciplinary health care team via HIE to improve communication between stakeholders, transitions in care, and resident health outcomes. Although resident access to HIE systems was not a significant predictor of total IT sophistication in our study, we see value in resident access to these systems. In order to understand how HIE systems could benefit nursing home staff, residents, and families, use cases have been developed to evaluate where HIE can have the most impact on communication and patient care [24]. Future studies should focus on the use of HIE by nursing home residents and resident representatives to better understand how these systems can be used to engage them in shared decision making, which is the cornerstone of person-centered care.

### Limitations

This paper reports on a secondary analysis of a national survey, and thus, response bias for nursing homes that choose not to participate in the survey should be considered a limitation. Some nursing homes may have chosen not to participate because they had no technology that could report higher overall IT sophistication than what actually exists. Analyses were limited to data available from the national IT sophistication survey; therefore, no direct measurement of resident or provider perceptions of access to technology were available. This study did not include measurement of health care outcomes, so it is not known whether residents’ access to technology facilitates self-management of health and health care. Finally, generalizability of findings is limited, and causality should not be implied as the result of this secondary analysis.

### Conclusions

Patient-facing technologies have only recently been introduced in postacute health care settings like nursing homes. Analyzing the extent to which residents have access to technology in a nationally representative sample is the first step toward understanding the benefits of and barriers to implementation. Engaging nursing home residents and their families through the use of technology has the potential to improve outcomes and promote person-centered care. However, to realize these potential improvements, we must learn more about how residents’ access to these technologies can be tailored for use in nursing homes and the perceived usefulness among various stakeholders including patients, family members, and their care teams.

## Abbreviations

IT: information technology
EHR: electronic health record
HIE: health information exchange
Total_IT*: total information technology sophistication
Res_Tech: resident access to technology

## References

[1] (2014). Centers for Disease Control and Prevention.

[2] (2018). Centers for Medicare and Medicaid Services.

[3] Wolf L, Harvell J, Jha AK (2012). Hospitals ineligible for federal meaningful-use incentives have dismally low rates of adoption of electronic health records. Health Aff (Millwood).

[4] Henry J, Pylypchuk Y, Searcy T, Patel V Office of the National Coordinator for Health Information Technology Data Brief - Health IT Dashboard.

[5] (2018). Office of the National Coordinator for Health Information Exchange - Health IT Dashboard.

[6] Frampton S, Guastello S National Academy of Medicine.

[7] (2014). National Quality Forum.

[8] Hill NL, Kolanowski AM, Milone-Nuzzo P, Yevchak A (2011). Culture change models and resident health outcomes in long-term care. J Nurs Scholarsh.

[9] (2018). HealthIT.gov.

[10] Gaskin S, Georgiou A, Barton D, Westbrook J (2012). Examining the role of information exchange in residential aged care work practices--a survey of residential aged care facilities. BMC Geriatr.

[11] Rantz MJ, Alexander G, Galambos C, Vogelsmeier A, Popejoy L, Flesner M, Lueckenotte A, Crecelius C, Zwygart-Stauffacher M, Koopman RJ (2014). Initiative to test a multidisciplinary model with advanced practice nurses to reduce avoidable hospitalizations among nursing facility residents. J Nurs Care Qual.

[12] Griffin A, Skinner A, Thornhill J, Weinberger M (2016). Patient Portals: Who uses them? What features do they use? And do they reduce hospital readmissions?. Appl Clin Inform.

[13] Mook PJ, Trickey AW, Krakowski KE, Majors S, Theiss MA, Fant C, Friesen MA (2018). Exploration of Portal Activation by Patients in a Healthcare System. Comput Inform Nurs.

[14] Alexander GL, Madsen RW, Miller EL, Schaumberg MK, Holm AE, Alexander RL, Wise KK, Dougherty ML, Gugerty B (2017). A national report of nursing home information technology: year 1 results. J Am Med Inform Assoc.

[15] (2019). Behind the scenes at nursing facilities. Post-acute And Long-term Medicine: A Pocket Guide (Current Clinical Practice).

[16] Centers for Medicare & Medicaid Services (2005). Medicare.gov.

[17] Alexander GL, Madsen R, Wakefield D (2010). A regional assessment of information technology sophistication in Missouri nursing homes. Policy Polit Nurs Pract.

[18] Alexander GL, Madsen R (2018). A National Report of Nursing Home Quality and Information Technology: Two-Year Trends. J Nurs Care Qual.

[19] Alexander GL, Wakefield DS (2009). Information technology sophistication in nursing homes. J Am Med Dir Assoc.

[20] Powell KR, Myers CM HIMSS: transforming health through information and technology.

[21] Kruse CS, Argueta DA, Lopez L, Nair A (2015). Patient and provider attitudes toward the use of patient portals for the management of chronic disease: a systematic review. J Med Internet Res.

[22] Pazinski S (2017). Health IT Buzz.

[23] Searcy T, Moriarty L (2016). Health IT Buzz.

[24] Alexander GL, Rantz M, Galambos C, Vogelsmeier A, Flesner M, Popejoy L, Mueller J, Shumate S, Elvin M (2015). Preparing Nursing Homes for the Future of Health Information Exchange. Appl Clin Inform.

